# Myeloid MKL1 Disseminates Cues to Promote Cardiac Hypertrophy in Mice

**DOI:** 10.3389/fcell.2021.583492

**Published:** 2021-04-09

**Authors:** Li Liu, Qianwen Zhao, Lin Lin, Guang Yang, Liming Yu, Lili Zhuo, Yuyu Yang, Yong Xu

**Affiliations:** ^1^Jiangsu Key Laboratory for Molecular and Medical Biotechnology, College of Life Sciences, Nanjing Normal University, Nanjing, China; ^2^Key Laboratory of Targeted Intervention of Cardiovascular Disease and Collaborative Innovation Center for Cardiovascular Translational Medicine, Department of Pathophysiology, Nanjing Medical University, Nanjing, China; ^3^Department of Pathology, Suzhou Municipal Hospital Affiliated with Nanjing Medical University, Suzhou, China; ^4^Department of Geriatrics, The Second Affiliated Hospital of Nanjing Medical University, Nanjing, China; ^5^Institute of Biomedical Research, Liaocheng University, Liaocheng, China

**Keywords:** transcriptional regulation, cardiac hypertrophy, macrophage, miRNA, NF-κB

## Abstract

Cardiac hypertrophy is a key pathophysiological process in the heart in response to stress cues. Although taking place in cardiomyocytes, the hypertrophic response is influenced by other cell types, both within the heart and derived from circulation. In the present study we investigated the myeloid-specific role of megakaryocytic leukemia 1 (MKL1) in cardiac hypertrophy. Following transverse aortic constriction (TAC), myeloid MKL1 conditional knockout (MFCKO) mice exhibit an attenuated phenotype of cardiac hypertrophy compared to the WT mice. In accordance, the MFCKO mice were protected from excessive cardiac inflammation and fibrosis as opposed to the WT mice. Conditioned media collected from macrophages enhanced the pro-hypertrophic response in cardiomyocytes exposed to endothelin in an MKL1-dependent manner. Of interest, expression levels of macrophage derived miR-155, known to promote cardiac hypertrophy, were down-regulated in the MFCKO mice compared to the WT mice. MKL1 depletion or inhibition repressed miR-155 expression in macrophages. Mechanistically, MKL1 interacted with NF-κB to activate miR-155 transcription in macrophages. In conclusion, our data suggest that MKL1 may contribute to pathological hypertrophy via regulating macrophage-derived miR-155 transcription.

## Introduction

Heart failure is defined as irreversible or permanent loss of rhythmic contraction and relaxation of the myocardium rendering insufficient supply of blood and oxygen to peripheral organs and tissues (Gaetani et al., 2020). Heart failure is one of the leading causes of non-accidental deaths worldwide (Udelson and Stevenson, 2016). A host of pathologies, including hypertension, infection, diabetes, and congenital structural heart disease, can cause heart failure. Regardless of the etiologies, heart failure is almost invariably preceded by cardiac hypertrophy, a process morphologically seen as an expansion in cross-sectional area of cardiomyocyte (Liu and Molkentin, 2016; Zhao et al., 2020). At the transcriptional level, cardiac hypertrophy is characterized by the re-activation of fetal genes (e.g., β-MHC). Generally perceived as a compensatory response attempting to preserve heart function under stress/injurious conditions, persistent hypertrophic response leads to maladaptation and eventually heart failure (Heineke and Molkentin, 2006).

Although the hypertrophic response takes place in the myocardium, it is hardly a cardiomyocyte-autonomous behavior. Instead, different cell types, including cardiac fibroblasts, endothelial cells, and circulating immune cells, contribute to the pathogenesis of pathological hypertrophy by forming cell-cell crosstalk with cardiomyocytes (Zhang et al., 2012; Frieler and Mortensen, 2015; Gogiraju et al., 2019). In addition, numerous humoral factors, originating from both intra-cardiac and extra-cardiac sources, act on the cardiomyocytes to regulate pathological hypertrophy (Ranjan et al., 2019). MicroRNAs or miRNAs, a group of ∼22 nt non-coding small RNAs, represent one of such factors (Patil et al., 2019; Dexheimer and Cochella, 2020). Transported via exosomes, miRNAs can transmit regulatory signals to cardiomyocytes from non-cardiomyocytes to regulate the hypertrophic response (Fan C. et al., 2020). Cardiac fibroblast derived miR-21-3p, for instance, promotes cardiomyocyte hypertrophy via targeting sorbin and SH3 domain-containing protein 2 (SORBS2) and PDZ and LIM domain 5 (PDLIM5) (Bang et al., 2014). Similarly, fibroblast derived miR-27a, miR-28-3p, and miR-34a promote oxidative stress, adverse cardiac remodeling, and heart failure in mice by targeting the antioxidant Nrf2 (Tian et al., 2018). On the contrary, a series of mesenchymal stem cell (MSC) derived miRNAs exert protective effects on cardiomyocytes to avert heart failure (Barile et al., 2014; Feng et al., 2014; Wang et al., 2015).

Megakaryocytic leukemia 1 (MKL1) is a transcriptional regulator with ubiquitous expression patterns (Wang et al., 2002). Developmentally redundant, MKL1 appears to play essential roles in a wide range of postnatal pathophysiological processes. MKL1 has been long considered as a key mechanosensor in various pathophysiological processes (Olson and Nordheim, 2010). Kuwahara et al. (2010) have reported that germline deletion of MKL1 in mice reduced the susceptibility to pressure overload induced cardiac hypertrophy. Further analysis revealed that MKL1 directly bound to the promoters of hypertrophic genes (e.g., atrial natriuretic peptide/ANP) in cultured cardiomyocytes in response to mechanical stretch and that MKL1 deficiency suppressed the expression of hypertrophic genes. Therefore, it was concluded that MKL1 might play a key role in the pathogenesis of pathological hypertrophy. However, whether the ability of MKL1 to regulate cardiac hypertrophy is cardiomyocyte-autonomous remains undetermined. In the present study, we report that MKL1 can contribute to the pathogenesis of cardiac hypertrophy by regulating myeloid-derived pro-hypertrophic cues.

## Materials and Methods

### Animals

All the animal experiments were reviewed and approved by the intramural Ethics Committee on Humane Treatment of Experimental Animals. Myeloid-specific deletion of MKL1 was achieved by crossing the *Mkl1*^*f/f*^ strain (Liu et al., 2018) with the *LyzM*-Cre strain, respectively (Yu et al., 2018). The offspring were designated based on genotyping: those with the *Mkl1*^*f/f*^; *LyzM*-Cre genotype (Cre positive) were called MFCKO and those with the *Mkl1*^*f/f*^ genotype (Cre negative) were called WT. Pathological cardiac hypertrophy was induced in mice by the transverse aortic constriction (TAC) procedure as previously described (Yu et al., 2015). Cardiac functions were evaluated by echocardiography (GE Vivid 7 equipped with a 14-MHz phase array linear transducer, S12, allowing a 150 maximal sweep rate). Mice were anesthetized using 1.5% isoflurane. The body temperature was maintained at 37^*o*^C using a heating pad.

### Cell Culture, Plasmids, Transient Transfection, and Reporter Assay

Murine macrophages RAW264.7 (ATCC) were maintained in DMEM supplemented with 10% FBS. Murine bone marrow-derived macrophages (BMDM) were isolated and cultured as described before (Yu et al., 2014). Neonatal rat ventricular myocytes (NRVM) were isolated and maintained as previously described (Yang et al., 2017). Endothelin (ET-1) was purchased from Peprotech. CCG-1423 (Chen et al., 2020a; Wu et al., 2020) and PDTC (Xu et al., 2017) were purchased from Selleck. MKL1 expression constructs and miR-155 promoter-luciferase constructs have been described previously (Basso et al., 2012; Thompson et al., 2013; Li et al., 2019c). Small interfering RNAs were purchased from Dharmacon. Transient transfection was performed with Lipofectamine 2,000. Cells were harvested 48 h after transfection and reporter activity was measured using a luciferase reporter assay system (Promega) as previously described (Yang et al., 2019a,b; Chen et al., 2020b,c; Li et al., 2020a).

### RNA Extraction and Real-Time PCR

RNA was extracted using an RNeasy RNA isolation kit (Qiagen) as previously described (Fan Z. et al., 2020). Reverse transcriptase reactions were performed using a SuperScript First-strand synthesis system (Invitrogen) as previously described (Li et al., 2020c; Lv et al., 2020; Mao et al., 2020; Yang et al., 2020). Real-time PCR reactions were performed on an ABI STEPONE Plus (Life Tech) with primers and Taqman probes purchased from Applied Biosystems. Ct values of target genes were normalized to the Ct values of housekeekping control gene (18s, 5′-CGCGGTTCTATTTTGTTGGT-3′ and 5′-TCGTCTTCGAAACTCCGACT-3′ for both human and mouse genes) using the ΔΔCt method and expressed as relative mRNA expression levels compared to the control group which is arbitrarily set as 1.

### Histology

Histological analysis was performed as previously described (Zhao et al., 2019; Dong et al., 2020; Li et al., 2020b). For immunofluorescence staining, antigen retrieval was performed by boiling the slides in sodium citrate (pH 6.0) for 1 min. The slides were washed 2 × 5 min in TBS plus 0.025% Triton X-100 with gentle agitation, blocked with 5% BSA, and incubated with anti-CD45 (Abcam, 1:200) overnight. After several washes with PBS, the slides were incubated with FITC-labeled secondary antibodies (Jackson, 1:200) for 30 min. DAPI (Sigma) was added and incubated with cells for 5 min prior to observation. Immunofluorescence was visualized on a confocal microscope (LSM 710, Zeiss). For quantification, stain-positive cells were counted on each slide and normalized to the control group which is arbitrarily set as 1. Images were quantified with ∼10 fields counted per mouse.

### Chromatin Immunoprecipitation

Chromatin Immunoprecipitation (ChIP) assays were performed essentially as described before (Fan Z. et al., 2019; Kong et al., 2019a,b; Li et al., 2019a,b,c,d,e; Liu et al., 2019; Lu et al., 2019; Shao et al., 2019; Weng et al., 2019; Sun et al., 2020). Briefly, chromatin was cross-linked with 1% formaldehyde. DNA was fragmented into ~500 bp pieces using a Branson 250 sonicator. Aliquots of lysates containing 200 μg of protein were used for each immunoprecipitation reaction with anti-MKL1 (Santa Cruz, sc-32909), anti-acetyl H3 (Millipore, 06-599), anti-trimethyl H3K4 (Millipore, 07-473), anti-NF-κB/RelA (Santa, Cruz, sc-372), and anti-BRG1 (Santa Cruz, sc-10768). Precipitated genomic DNA was amplified by real-time PCR. A total of 10% of the starting material is also included as the input. Data are then normalized to the input and expressed as% of recovery.

### Statistical Analysis

One-way ANOVA with *post hoc* Scheffe analyses were performed using an SPSS package. Unless otherwise specified, *P*-values smaller than 0.05 were considered statistically significant.

## Results

### MKL1 Deletion in Myeloid Cells Attenuates Pathological Cardiac Hypertrophy in Mice

**FIGURE 1:**
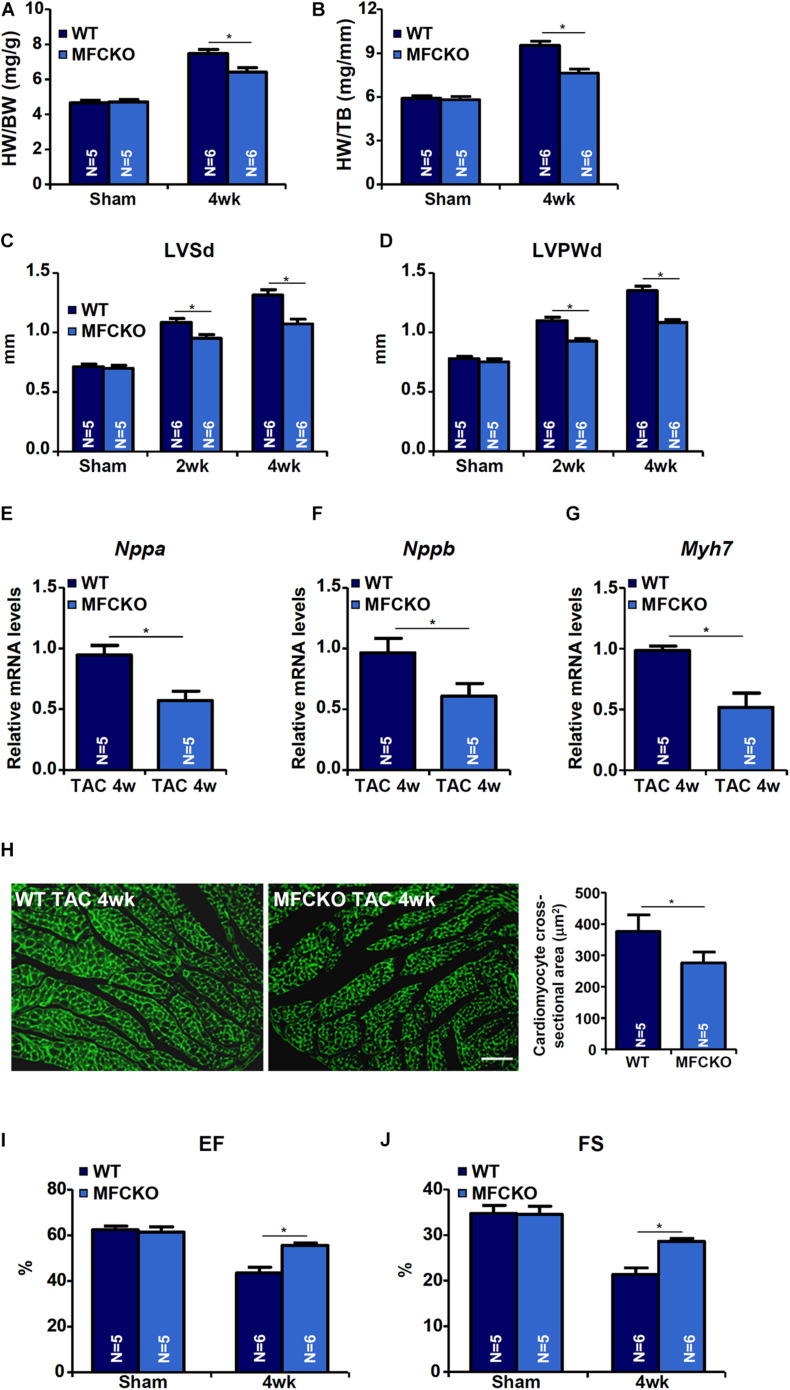
MKL1 deletion in macrophages attenuates pathological cardiac hypertrophy in mice. Myeloid-specific MKL1 knockout mice (MFCKO) and wild type (WT) littermates were subjected to the TAC procedure or the sham procedure. **(A)** Heart weight versus body weight. **(B)** Heart weight versus tibia bone length. **(C)** Left ventricular systolic diameter (LVSd) values. **(D)** Left ventricular posterior wall diameter (LVPWd) values. **(E–G)** Hypertrophic genes were measured by qPCR. **(H)** Cardiomyocyte cross-section areas were measured by wheat germ agglutinin (WGA) staining and quantified by Image J. **(I)** Ejection fraction (EF) values. **(J)** Fractional shortening (FS) values. *N* = 5∼6 mice. Data represent mean ±SD. *p < 0.05, two-tailed *t*-test.

Macrophages play key roles in the pathogenesis of cardiac hypertrophy (Schiattarella and Hill, 2015). To evaluate whether MKL1 deficiency in macrphages would impact pathological hypertrophy, the TAC procedure was performed in myeloid conditional MKL1 knockout (MFCKO) mice and wild type (WT) littermates. The deletion of MKL1 in myeloid cells was verified by Western blotting (Supplementary Figure 1). Measurements of heart weight/body weight ratios (Figure 1A), heart weight/tibia bone length ratios (Figure 1B), left ventricular systolic diameter (LVSd) values (Figure 1C), left ventricular posterior wall diameter (LVPWd) values (Figure 1D), qPCR examination of atrial natriuretic peptide (ANP, Figure 1E), brain natriuretic peptide (BNP, Figure 1F), and myosin heavy chain beta isoform (β-MHC, Figure 1G) in the heart, and wheat germ agglutinin (WGA, which detects cell membrane components such as N-acetylglucosamine and N-acetylneuraminic acid) staining of cardiomyocyte cross-sectional areas (Figure 1H) all indicated that pressure overload induced cardiac hypertrophy was attenuated in MFCKO mice compared to WT mice. At 4 week after the surgical procedure, heart functions, as indicated by left ventricular ejection fraction (EF) values and fractional shortening (FS) values, were preserved better in MFCKO mice than in WT mice (Figures 1I,J).

### MKL1 Deletion in Myeloid Cells Ameliorates Pressure Overload Induced Cardiac Inflammation and Fibrosis in Mice

**FIGURE 2:**
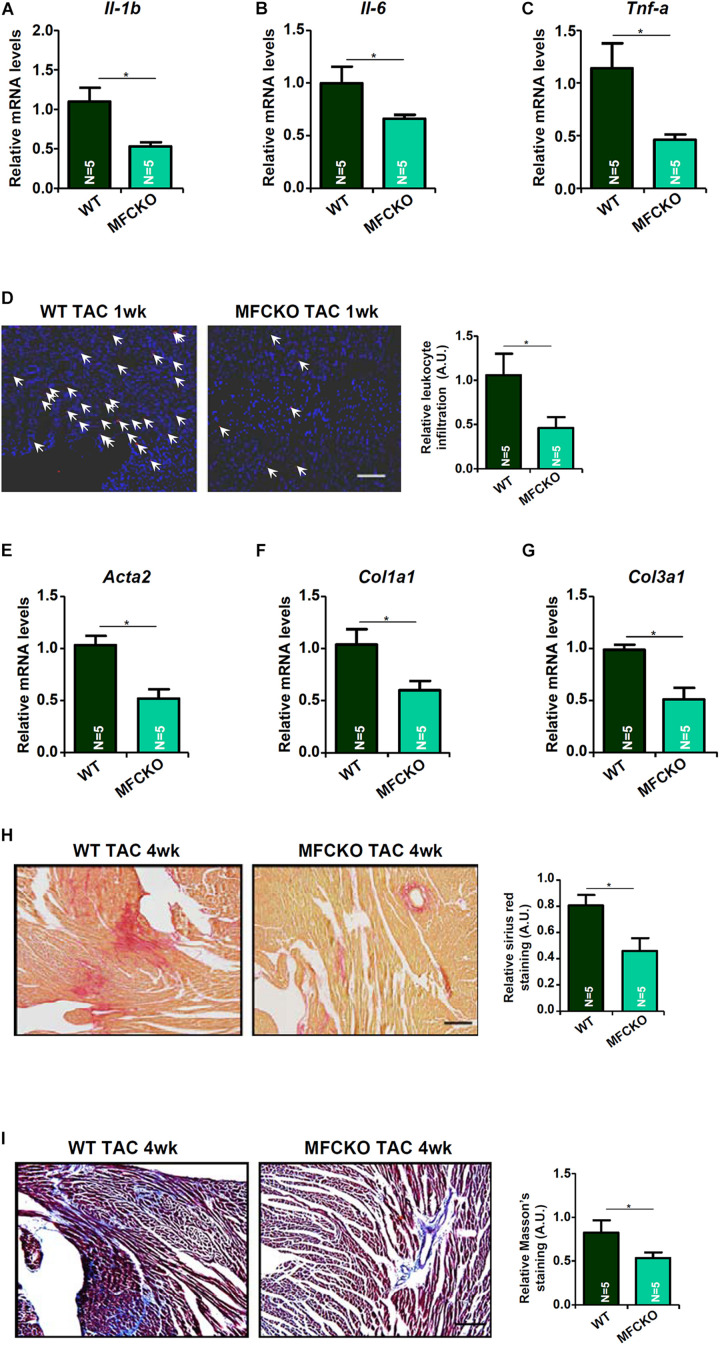
MKL1 deletion in macrophages ameliorates pressure overload induced cardiac inflammation and fibrosis in mice. MFCKO mice and WT mice were subjected to the TAC procedure. **(A–C)** Pro-inflammatory genes were measured by qPCR. **(D)** CD45 staining. **(E–G)** Pro-fibrogenic genes were measured by qPCR. **(H)** Picrosirius red staining. **(I)** Masson’s trichrome staining. *N* = 5 mice for each group. Data represent mean ± SD. *p < 0.05, two-tailed *t*-test.

Next, we analyzed the effects of myeloid-specific MKL1 deletion on cardiac inflammation and cardiac fibrosis during the pathogenesis of cardiac hypertrophy in mice. Quantitative PCR showed that expression levels of pro-inflammatory cytokines, including IL-1β (Figure 2A), IL-6 (Figure 2B), and TNF-α (Figure 2C), were lower in the MFCKO mice than in the WT mice. Suppression of cardiac inflammation as a result of MKL1 loss in myeloid cells was confirmed by immunofluorescence staining of CD45^+^ cells showing that there were much fewer immune infiltrates in the MFCKO hearts than the WT hearts (Figure 2D). On the other hand, qPCR analysis demonstrated that cardiac expression of pro-fibrogenic genes, including α-SMA (Figure 2E), collagen type I (Figure 2F), and collagen type III (Figure 2G), was down-regulated in the MFCKO mice compared to the WT mice. Picrosirius red staining (Figure 2H) and Masson’s trichrome staining (Figure 2I) both confirmed that cardiac fibrosis was mitigated by myeloid-specific deletion of MKL1 in mice. Taken together, these data suggest that the pro-hypertrophic ability of MKL1 may originate from macrophages.

### MKL1 Regulates a Pro-Hypertrophic Cue From Macrophages

**FIGURE 3:**
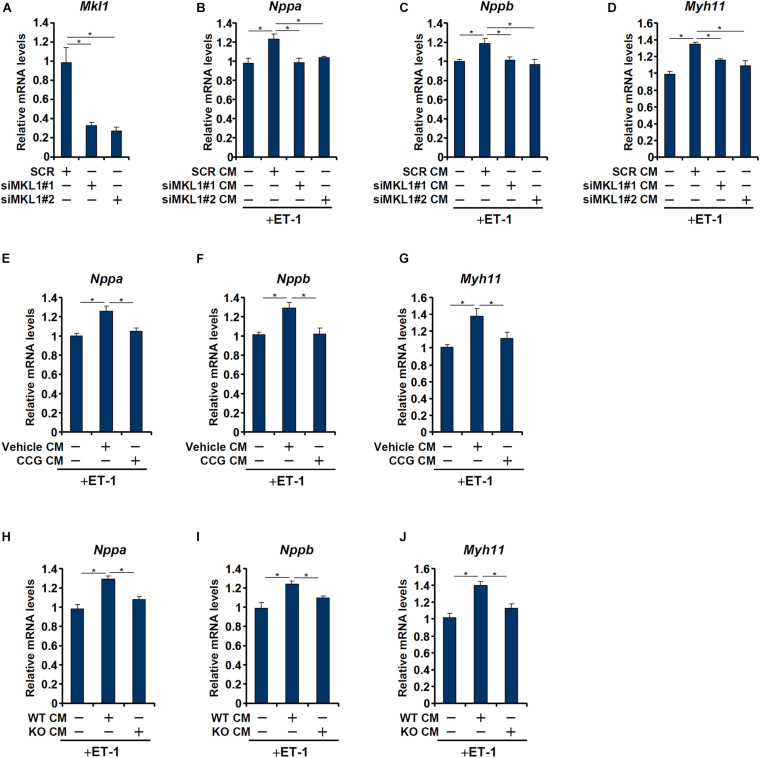
MKL1 regulates a pro-hypertrophic cue from macrophages. **(A–D)** RAW cells were transfected with siRNA targeting MKL1 or scrambled siRNA (SCR). Conditioned media were collected 48 h after transfection and applied to primary neonatal rat ventricular myocytes along with endothelin. Knockdown efficiencies were verified by qPCR and Western **(A)**. ANP **(B)**, BNP **(C)**, and β-MHC **(D)** levels were examined by qPCR. **(E–G)** RAW cells were treated with or without CCG-1423 (10 μM) for 24 h. Conditioned media were collected 48 h after transfection and applied to primary neonatal rat ventricular myocytes along with endothelin. ANP **(E)**, BNP **(F)**, and β-MHC **(G)** levels were examined by qPCR. **(H–J)** Conditioned media were collected from WT and MFCKO bone marrow derived macrophages and applied to primary neonatal rat ventricular myocytes along with endothelin. ANP **(E)**, BNP **(F)**, and β-MHC **(G)** levels were examined by qPCR. Data represent mean ± SD. *p < 0.05, One-way ANOVA with *post hoc* Scheffe test.

Having determined that macrophage MKL1 plays an essential role in the development of pressure overload induced pathological hypertrophy in mice, we hypothesized that an MKL1-dependent pro-hypertrophic cue may be transmitted from macrophages to cardiomyocytes. To this send, conditioned media (CM) were collected from cultured macrophages (RAW) and applied to freshly isolated neonatal rat left ventricular myocytes (NRVM) in the presence of ET-1, a potent pro-hypertrophic factor. The addition of macrophage CM enhanced the pro-hypertrophic effects of ET-1 as judged by the expression levels of ANP (Figure 3B), BNP (Figure 3C), and β-MHC (Figure 3D). MKL1 depletion (Figure 3A for knockdown efficiency) in macrophages, however, severely compromised the potency of the CM to potentiate ET-1 induced hypertrophy of NRVM. Similarly, pre-treatment of macrophages with CCG-1423, a small-molecule MKL1 inhibitor, significantly dampened the production of the pro-hypertrophic signal (Figures 3E–G). Finally, it was observed that CM collected from WT bone marrow derived macrophages (BMDMs) had a much stronger pro-hypertrophic effect than those from MKL1 KO BMDMs (Figures 3H–J).

### MKL1 Regulates miR-155 Expression in Macrophages

**FIGURE 4:**
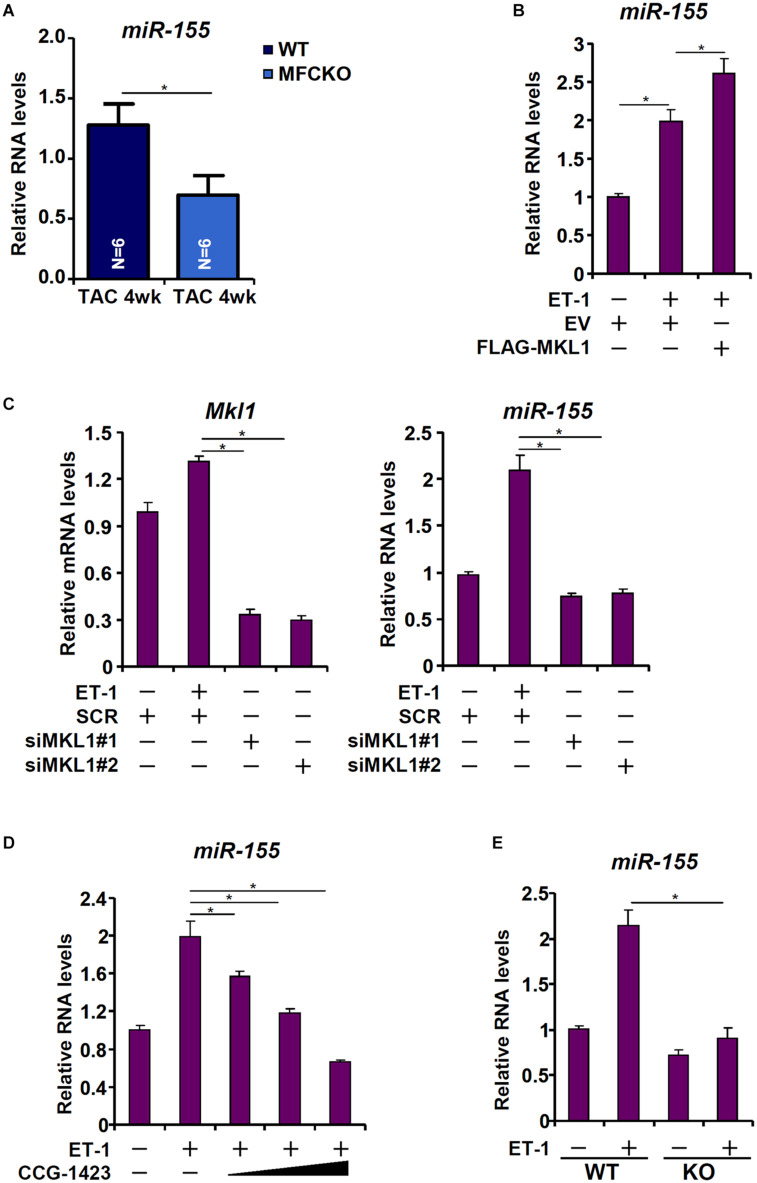
MKL1 regulates miR-155 expression in macrophages. **(A)** MFCKO mice and WT mice were subjected to the TAC procedure. miR-155 levels were examined by qPCR. *N* = 5 mice for each group. Data represent mean ± SD. *p < 0.05, two-tailed *t*-test. **(B)** RAW cells were transfected with MKL1 expression construct or an empty vector (EV) followed by treatment with endothelin. miR-155 levels were examined by qPCR. **(C)** RAW cells were transfected with siRNA targeting MKL1 or SCR followed by treatment with endothelin. miR-155 levels were examined by qPCR. **(D)** RAW cells were treated with endothelin and CCG-1423. miR-155 levels were examined by qPCR. **(E)** BMDMs isolated from WT and MFCKO were treated with endothelin. miR-155 levels were examined by qPCR. Data represent mean ± SD. *p < 0.05, One-way ANOVA with *post hoc* Scheffe test.

Heymans et al. (2013) have reported that macrophage-derived miR-155 is essential for the development of pressure overload induced pathological hypertrophy in mice. We therefore postulated that MKL1 might regulate miR-155 expression in macrophages. Indeed, miR-155 levels were decreased in the MFCKO hearts compared to the WT hearts (Figure 4A). Over-expression of MKL1 potentiated the induction of miR-155 expression by ET-1 treatment in RAW cells (Figure 4B). Next, endogenous MKL1 was silenced in RAW cells; MKL1 knockdown dampened the induction of miR-155 by ET-1 (Figure 4C). Likewise, inhibition of MKL1 activity by CCG-1423 suppressed the induction of miR-155 by ET-1 (Figure 4D). Comparison of miR-155 expression levels in WT BMDMs and MKL1 KO BMDMs confirmed that MKL1 played an essential role in regulating miR-155 expression in macrophages (Figure 4E).

### MKL1 Interacts With NF-κB to Activate miR-155 Transcription in Macrophages

**FIGURE 5:**
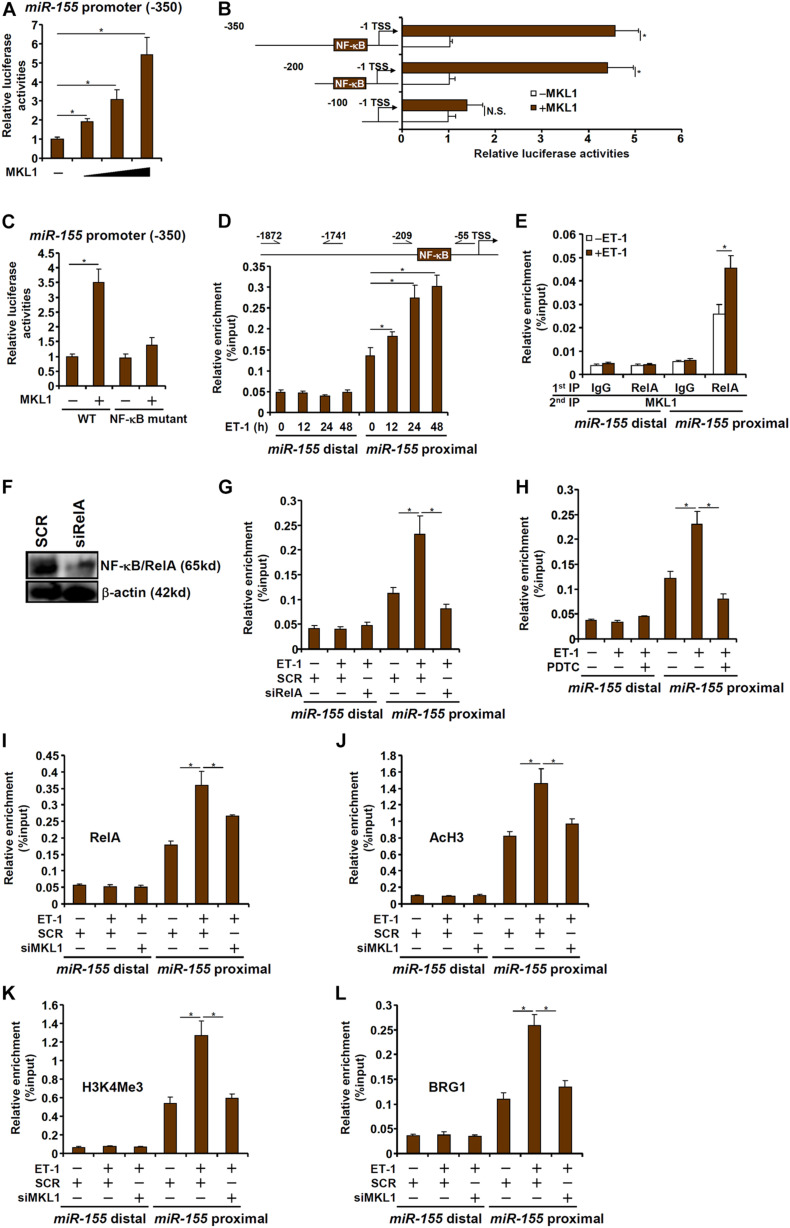
MKL1 interacts with NF-κB to activate miR-155 transcription in macrophages. **(A)** A miR-155 promoter-luciferase construct was transfected into HEK293 cells with or without MKL1. Luciferase activities were normalized by both protein concentration and GFP fluorescence. **(B)** miR-155 promoter-luciferase constructs of various lengths were transfected into HEK293 cells with or without MKL1. Luciferase activities were normalized by both protein concentration and GFP fluorescence. **(C)** Wild type or NF-κB site mutant miR-155 promoter-luciferase construct was transfected into HEK293 cells with or without MKL1. Luciferase activities were normalized by both protein concentration and GFP fluorescence. **(D)** RAW cells were treated with endothelin and harvested at indicated time points. ChIP assays were performed with anti-MKL1. **(E)** RAW cells were treated with or without endothelin for 24h. Re-ChIP assays were performed with indicated antibodies. **(F,G)** RAW cells were transfected with siRNA targeting NF-κB or SCR followed by treatment with endothelin. Knockdown efficiencies and were verified by Western. ChIP assays were performed with anti-MKL1. **(H)** RAW cells were treated with endothelin and/or PDTC. ChIP assays were performed with anti-MKL1. **(I–L)** RAW cells were transfected with siRNA targeting MKL1 or SCR followed by treatment with endothelin. ChIP assays were performed with anti-RelA **(I)**, anti-acetyl H3 **(J)**, anti-H3K4Me3 **(K)**, and anti-BRG1 **(L)**. Data represent mean ± SD. *p < 0.05, One-way ANOVA with *post hoc* Scheffe test.

To investigate whether the regulation of miR-155 expression by MKL1 occurred at the transcriptional level, a miR-155 promoter-luciferase construct (-350/-1) was transfected into HEK293 cells. MKL1 over-expression dose-dependently activated the miR-155 promoter (Figure 5A), indicating that MKL1 may indeed directly regulate miR-155 transcription. Next, a series of truncated miR-155 promoter-luciferase constructs were transfected into HEK293 cells with or without MKL1 to determine the region where MKL1 may potentially bind. As shown in Figure 5B, MKL1 was able to activate the miR-155 promoter only in the presence of an intact NF-κB site. When the NF-κB site was mutated, MKL1 lost the ability to activate the miR-155 promoter (Figure 5C). ChIP assay demonstrated that there was significant MKL1 binding within the proximal miR-155 promoter surrounding the NF-κB site, which was further augmented by ET-1 treatment; in contrast, no appreciable MKL1 binding was detected on the distal miR-155 promoter (Figure 5D). Re-ChIP assay confirmed that a RelA-MKL1 complex was detectable on the proximal, but not the distal, miR-155 promoter (Figure 5E). Either RelA knockdown (Figures 5F,G) or RelA inhibition (Figure 5H) by a small-molecule compound (PDTC) abrogated the binding of MKL1, suggesting that MKL1 may rely on NF-κB/RelA to be recruited to the miR-155 promoter. Of interest, MKL1 knockdown weakened the binding of RelA to the miR-155 promoter (Figure 5I). This was likely due to an altered chromatin structure because MKL1 deficiency reduced the levels of acetyl H3 (Figure 5J) and trimethyl H3K4 (Figure 5K) and abolished the recruitment of chromatin remodeling protein BRG1 (Figure 5L) on the miR-155 promoter. We thus conclude that interplay between MKL1 and NF-κB/RelA contributes to miR-155 transcription in macrophages.

## Discussion

**FIGURE 6:**
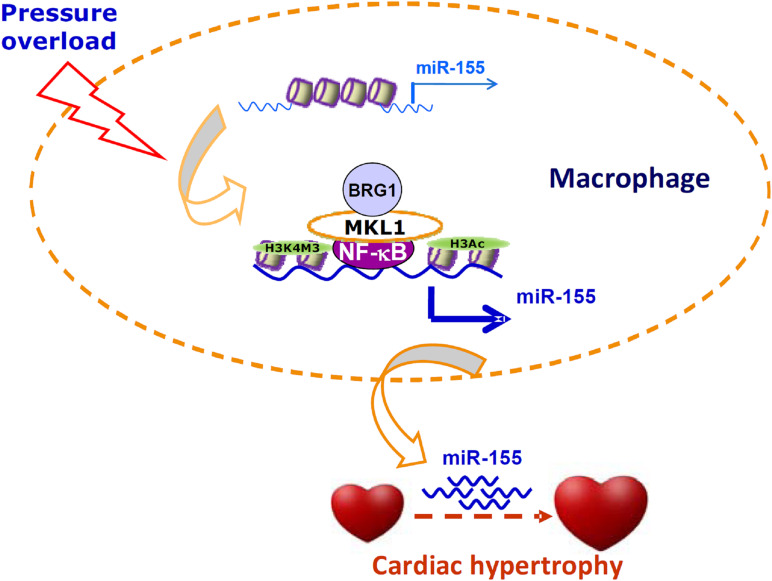
A schematic model. In response to pressure overload, MKL1 is recruited to the miR-155 promoter by interacting with NF-κB in macrophages. MKL1 recruitment leads to altered histone modifications and active chromatin remodeling stabilizing NF-κB binding and activating miR-155 transcription. miR-155, likely transmitted via exosomes, acts on the cardiomyocytes to promote hypertrophy.

Cell–cell crosstalk represents an important mechanism that guards the physiological and functional integrity of the heart but at the same time underlies the pathogenesis of cardiovascular diseases including pathological hypertrophy and heart failure (Tirziu et al., 2010). Traditionally, cardiac macrophages are thought to contribute to disturbances of cardiomyocyte function by producing/releasing inflammatory cytokines and reactive oxygen species. However, recent studies have found that macrophages may influence cardiomyocyte conductance and regeneration (Gomez et al., 2018). Here we report that MKL1 may indirectly regulate cardiomyocyte hypertrophy by controlling the production of a pro-hypertrophic cue (miR-155) in macrophages (Figure 6).

Kuwahara et al. (2010) have shown that mice with germ line deletion of MKL1 are resistant to pressure overload induced cardiac hypertrophy; it was not determined whether the ability of MKL1 to modulate the hypertrophic response is cardiomyocyte autonomous. A recent report showed that TAC-induced cardiac hypertrophy is equivalent in mice with a systemic deletion of MKL1 and a simultaneous cardiomyocyte conditional deletion of its closest sibling MKL2 (also known as MRTF-B) and in WT mice (Trembley et al., 2018). Unlike MKL2, MKL1 is dispensable for embryonic development (Oh et al., 2005; Sun et al., 2006). Although both MKL1 and MKL2 exhibit similar tissue/cell distribution, MKL1 has been reported to function as a much stronger transcriptional activator than MKL2 in cultured cells (Fang et al., 2011; Yang et al., 2013, 2014; Yu et al., 2014, 2017). It is not clear at this point how the loss of MKL1/MKL2 is tolerated during cardiac hypertrophy. One possibility is that myocardin, the founding member of the MRTF family, becomes hyper-activated and mediates pressure overload induced pathological hypertrophy (Xing et al., 2006).

Our data suggest that myeloid conditional MKL1 knockout (MFCKO) mice were more resistant to the development of cardiac hypertrophy than WT mice (Figure 1). This is consistent with our recent finding that myeloid MKL1, but not cardiomyocyte MKL1, contributes to cardiac ischemia-reperfusion injury in mice (Yu et al., 2018). This also strongly argues for the indispensable role of MKL1 in regulating macrophage phenotype and function to promote disease pathogenesis when combined with previous reports indicating that MKL1 contributes to colitis (Yu et al., 2014), atherosclerosis (Minami et al., 2012), and sepsis (Yu et al., 2017). We further demonstrate that MKL1 activated the transcription of miR-155, a macrophage-derived pro-hypertrophic cue (Heymans et al., 2013), by interacting with NF-κB/RelA. Again, this piece of evidence echoes previous findings, based on analysis of single gene expression (Fang et al., 2011; Yang et al., 2014; Chen et al., 2015) and genomewide expression (Xie, 2014; Yu et al., 2017), that transcription regulation by MKL1 in macrophage is often considered an extension of its interaction with NF-κB/RelA. Congruently, several reports suggest that NF-κB inhibition suppresses cardiac hypertrophy although a direct role of macrophage RelA has yet to be established *in vivo* (Andersen et al., 2012; Gaspar-Pereira et al., 2012). It should be pointed out that we do not propose miR-155 as the sole macrophage-derived, MKL1-dependent pro-hypertrophic cue. We have previously shown that MKL1 is a transcriptional activator of matrix metalloproteinase 9 (MMP9) in lung cancer cells (Cheng et al., 2015). Coincidently, macrophage-specific over-expression of MMP9 significantly exacerbates cardiac hypertrophy in mice (Toba et al., 2017). In addition, other transcriptional targets of MKL1 including inflammatory cytokines, chemokines, and oxidants may contribute to cardiac hypertrophy.

There are a few lingering issues that deserve further attention. First, although we propose that miR-155 derived from macrophages is directly regulated by MKL1 and may contribute to the pro-hypertrophic response in cardiomyocytes. An alternative scenario taking place in cardiomyocytes could equally contribute to cardiac hypertrophy because miR-155 is expressed and can be up-regulated by pro-hypertrophic stimuli in cardiomyocytes (Seok et al., 2014). It has also been shown that ablation of miR-155 in cardiomyocytes appears to be sufficient to blunt the hypertrophic response induced by phenoephrine (PE) (Seok et al., 2014). This alternative model wherein MKL1 activates miR-155 transcription in cardiomyocytes to promote cardiac hypertrophy is certainly tempting in light of our recent report that cardiomyocyte-specific MKL1 deletion attenuates angiotensin II induced cardiac hypertrophy in mice (Wu et al., 2020). Second, the finding that MKL1 relies on NF-κB to regulate miR-155 transcription reinforces the notion that MKL1 and NF-κB are functionally interconnected given previous investigations linking these two factors in the pathogenesis of atherosclerosis (Fang et al., 2011), colitis (Yu et al., 2014), and septic shock (Yu et al., 2017), However, the precise role of NF-κB in the pathogenesis of cardiac hypertrophy has not been conclusively demonstrated especially in animal models likely due to the isoform-specific effects of NF-κB (Gordon et al., 2011). In addition, no direct evidence exists to demonstrate the influence of myeloid-specific NF-κB manipulation on cardiac hypertrophy. Therefore, our observation that MKL1 interacts with NF-κB to activate miR-155 transcription in macrophages cannot be construed as proof of myeloid NF-κB regulating cardiac hypertrophy. Instead, further investigations are needed to disentangle the functional overlap of NF-κB and MKL1 in regulating macrophage behavior and its implication *in vivo*.

In summary, we present evidence to show that myeloid MKL1 plays an essential role in the pathogenesis of cardiac hypertrophy. Previously, we have shown that endothelial MKL1 contributes to cardiac hypertrophy by activating ET-1 transcription (Weng et al., 2015a,b). These data collectively suggest that MKL1 regulates cardiac hypertrophy, at least in part, in a non-autonomous manner.

## Data Availability Statement

The original contributions presented in the study are included in the article/Supplementary Material, further inquiries can be directed to the corresponding author/s.

## Ethics Statement

The animal study was reviewed and approved by the Nanjing Normal University Ethics Committee on Humane Treatment of Experimental Animals.

## Author Contributions

YY and LZ conceived the project. LLu, QZ, LLn, GY, and LY designed and performed experiments and collected and analyzed the data. YX wrote the manuscript. YY and LZ secured funding and provided supervision. All authors contributed to the article and approved the submitted version.

## Conflict of Interest

The authors declare that the research was conducted in the absence of any commercial or financial relationships that could be construed as a potential conflict of interest.
