# Supplementary material for: Myeloid MKL1 Disseminates Cues to Promote Cardiac Hypertrophy in Mice
Source: Front Cell Dev Biol. 2021 Apr 9;9:583492. doi: 10.3389/fcell.2021.583492 (PMC8063155; doi:10.3389/fcell.2021.583492)
Supplement: Supplementary file 1 [file Data_Sheet_1.PDF]

Liu L et al: Myeloid MKL1 disseminates cues to promote cardiac hypertrophy in mice
Online supplementary material

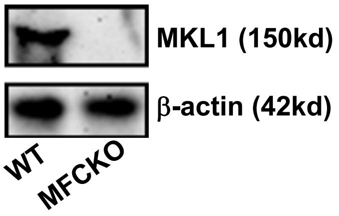

Fig.S1: Bone marrow derived macrophages were isolated from WT and MKL1 MFCKO mice. MKL1 expression levels were examined by Western blotting.
